# Legal Intelligence

**Published:** 1899-05-27

**Authors:** 


### LEGAL INTELLIGENCE.

### OBSTRUCTING REMOVAL OF PATIENTS TO FEVER HOSPITALS.

There is a class of people who seem to think it their bounden duty to oppose the execution of all sanitary laws, and who regard the officials whose duty it is to administer them as their personal enemies. These persons are unable to appreciate the importance to the community of which they form a part of prompt action in dealing with infectious diseases, and therefore they will in defence of their own supposed rights and privileges do their best to render nugatory the whole purpose of our public health laws. It is eminently satisfactory that there exists a means of disarming opposition of this kind, and the Queen's Bench Division, in the case of the Queen v. Davey, have done a distinct public service in explaining the law. The facts were shortly as follows. A magistrate, acting upon the certificate of a medical practitioner, had made an order for the removal of a child suffering from scarlet fever to a hospital. The mother resisted the order and prevented the officers from carrying out their order, alleging that the hospital in question was not a suitable one. Whatever the merits or demerits of a particular institution, there clearly is no justification for forcibly resisting the law. The action of the mother led to proceedings being instituted against her under the Public Health Act for obstructing the execution of the order, but the justices, apparently thinking they were at liberty to go behind the order of removal and to inquire into the circumstances under which it was made, refused to convict. Therefore a mandamus was applied for to compel the justices to state a case for the opinion of the High Court, it being contended that the justices could not go behind the order for removal, and must deal solely with the case of obstruction to its execution. The Queen's Bench Division held that this was the proper view of the case, and they dwelt strongly on the danger to the public which would result if orders for removal could be made the subject of argument. In fact, they showed that the whole legislation would be useless if persons could obstruct and delay the operation of the order, which was intended by the legislature to be a summary remedy for the protection of the public from infectious diseases. In such cases promptness of action was essential. At the same time there might be instances where removal would be wrong, however much a medical officer of health might desire it, and it is specially to prevent the sanitary authority, through its servants, exercising undue pressure or doing an injury to any individual in the execution of its powers that the Act makes it necessary to obtain an order of removal from a magistrate, who, before making it should satisfy himself as to the conditions, viz., that a medical certificate has been obtained and that there is a suitable hospital within a convenient distance and no proper accommodation at the patient's home. The remedy in case of an improper order having been made would be, not to obstruct the execution of it, but to proceed probably by writ of *certiorari* or *habeas corpus* to question its validity. If the allegations of the mother in the present case as to the shortcomings of the hospital were properly put forward and substantiated, no doubt she could have the removal order reviewed. In the present case she took the wrong course entirely, and the result of the proceedings is to show that such opposition as she made to a most salutary measure of public protection is entirely indefensible.

